# Quantum computing applications in drug discovery

**DOI:** 10.1093/bib/bbag274

**Published:** 2026-05-31

**Authors:** Jing Li, Leyi Wei, Henry H Y Tong, Quan Zou

**Affiliations:** Department of Microbiology, University of Hong Kong, 19/F, Block T, Queen Mary Hospital, 102 Pokfulam Road, Pokfulam, Hong Kong, China; Centre of AI-driven Drug Discovery, Faculty of Applied Sciences, Macao Polytechnic University, R. de Luís Gonzaga Gomes, Macao, 999078, China; Centre of AI-driven Drug Discovery, Faculty of Applied Sciences, Macao Polytechnic University, R. de Luís Gonzaga Gomes, Macao, 999078, China; Faculty of Health Sciences and Sports, Macao Polytechnic University, R. de Luís Gonzaga Gomes, Macao, 999078, China; Centre of AI-driven Drug Discovery, Faculty of Applied Sciences, Macao Polytechnic University, R. de Luís Gonzaga Gomes, Macao, 999078, China; Institute of Fundamental and Frontier Sciences, University of Electronic Science and Technology of China, No. 4, Section 2, North Jianshe Road, Chengdu, 610054, China

**Keywords:** quantum computing, drug discovery, deep learning–based virtual screening, molecular docking-based virtual screening, molecular dynamics, hybrid quantum–classical workflows, Noisy Intermediate-Scale Quantum

## Abstract

In early drug discovery, virtual screening based on deep learning, virtual screening based on molecular docking, and molecular dynamics are three widely used computational strategies, but they always face a trade-off between throughput, search stability, and physical fidelity. This article discusses how quantum computing can be integrated into these processes under the constraints of Noisy Intermediate-Scale Quantum (NISQ). At present, the most realistic role of quantum computing is not the complete replacement of classical processes, but modular coprocessing for selected decision-sensitive subroutines. In the screening of deep learning, quantum modules are mainly inserted into selected components of the model. In predictive models, they are used to enhance representation learning or feature extraction. In generative models, they serve as priors or generators. In docking screening, quantum integration is suitable for specific substeps such as site recognition, pose search, and flexible docking. In molecular dynamics, representative examples include ground state *ab initio* molecular dynamics, annealer-based trajectory propagation, and excited state molecular dynamics, while most large-scale sampling is still done by classical methods. The actual problem in these scenarios is not whether the quantum module can be inserted, but whether it can provide repeatable gains related to decision-making under the constraints of actual running time and resources. Therefore, we emphasize strong classical baselines, reliable ranking and calibration, transparent resource reporting, and evaluation at downstream decision points as key criteria for assessing progress in the near term.

## Introduction

### Current status and challenges in drug discovery

#### General context of drug discovery and development

**Figure 1:**
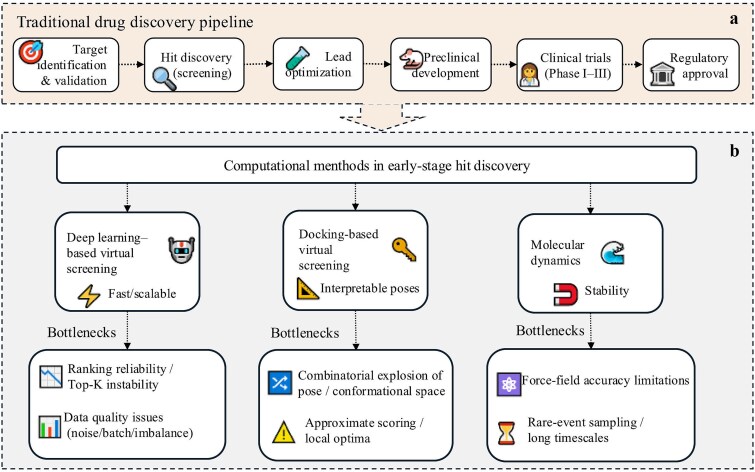
Background of drug discovery and representative calculation methods in early screening.

Modern drug research and discovery can be broadly divided into two major phases: discovery and development. The discovery phase encompasses target identification and validation, as well as lead identification and optimization, while the development phase involves systematic preclinical evaluation, process and formulation development, and subsequent validation through human trials. Despite these structured stages, the drug development pipeline typically spans 10–15 years and often exceeds USD 1–2 billion in cost, with high attrition rates resulting in only a small fraction of candidates ultimately reaching the market [1]. Among the multiple stages of drug discovery and development, early stage computational methods are particularly important for supporting candidate discovery and refinement. In this context, computational methods are widely used to improve efficiency and guide experimental testing [2–5] (Fig. 1a).

#### Role of computational methods in drug discovery

With the rapid expansion of target structural data, screening datasets, and chemical space, computer-aided drug design (CADD) has become an indispensable component of early stage drug discovery. By shifting much of the trial and error process to *in silico* workflows, CADD reduces experimental cost while improving hit identification rates [6]. In recent years, machine learning and deep learning have further enhanced the rapid evaluation and prioritization of candidate molecules. Consequently, virtual screening has evolved from approaches based on rules and scoring functions toward direct prediction driven by data, enabling efficient prefiltering of extremely large compound libraries (Fig. 1b).

In early stage drug discovery, three representative computational roles are frequently encountered: rapid screening, pose generation, and dynamical assessment. Deep learning–based virtual screening takes as input molecular fingerprints, simplified molecular input line entry system (SMILES) strings, molecular graphs, and 3D geometric features. These models learn mappings from molecular structure to activity, binding affinity, or phenotypic response, and then perform batch inference to rank large compound libraries. Its key advantages are scalability and speed, which make it well suited for first-pass filtering across vast chemical spaces. The second category is molecular docking-based virtual screening, which generates plausible ligand binding poses within a defined binding pocket and applies scoring functions to obtain coarse estimates of binding affinity. Molecular docking is widely used to generate interpretable binding hypotheses [7–11] (Fig. 1b). The third category is molecular dynamics, which simulates the time evolution of molecular complexes, typically in explicit or implicit solvent depending on the model and application, thereby enabling the assessment of structural stability and changes in key interactions. Molecular dynamics is often used to evaluate the physical plausibility of docking poses and to analyse induced conformational changes. In a specific system, molecular dynamics simulation (sometimes combined with free energy methods) can compare candidate compounds more accurately. However, this advantage usually depends on the specific situation and is not universally observed in all target or drug discovery environments (Fig. 1b). In some projects, virtual screening based on deep learning, virtual screening based on docking, and molecular dynamics can be used in stages or complement each other [12–15]. However, these methods are not intrinsically sequential and do not always need to be used together. Depending on the specific task, goal, and available resources, each method can be used as an independent computing entrance.

In many drug discovery projects, the choice of the final compound is not simply determined by the calculation ranking. More commonly, candidate compounds with higher rankings will be further screened by drug chemists and project teams based on factors such as synthesizability, novelty, developability, accessibility of synthesis, and target specificity knowledge before entering experimental verification. In this context, the calculation score should be regarded as one of the reference factors for project level decision-making, rather than an independent final decision-making rule. From this perspective, the actual value of quantum computing is unlikely to replace human judgment, but plays a role by improving the reliability of ranking and decision-making confidence in screening under expert guidance.

### Accuracy and throughput trade-offs in computational drug discovery

Although informatics and computational methods have significantly improved the efficiency of early screening, the overall speed of drug research and development has not improved accordingly. One of the fundamental reasons is that there is always a trade-off between accuracy and scale. Higher physical fidelity usually means higher computational costs, and higher flux often depends on stronger approximations, which may introduce additional uncertainty [16, 17]. This contradiction is particularly prominent in the three key calculation methods introduced in this article: virtual screening based on deep learning, virtual screening based on molecular docking, and a molecular dynamics simulation.

#### Speed and reliability limits in virtual screening based on deep learning

The deep learning model is good at high-throughput reasoning of massive chemical libraries, so it can quickly screen compounds at a lower cost. But its limitations are also obvious. This kind of model mainly captures the patterns in the training data. When applied to new chemical skeletons, different detection conditions, or unfamiliar target categories, it will be affected by distribution shift and reduce performance. In addition, labeling datasets usually contain experimental noise, batch effects, and category imbalance deviations, which reduce the comparability and interpretability of scores [18, 19]. Therefore, when screening large compound libraries, deep learning is more suitable as a tool for rough screening and sorting, rather than for final decision-making. Molecular docking, molecular dynamics simulation, or experimental verification are still necessary means for priority sorting [20–22] (Fig. 1b).

#### Interpretability and stability limits in virtual screening based on molecular docking

The main task of molecular docking is to search for possible ligand poses in the binding pocket within a high-dimensional conformational space and rank them using scoring functions. The main computing cost comes from numerous sampling and repeated scoring. Accuracy is still a key limiting factor because many scoring functions rely on simplified assumptions about solvents, receptor flexibility, induced fit, and entropy. Differences across software packages and parameter settings can further amplify the uncertainty in screening results [23]. Therefore, molecular docking is more suitable to be regarded as a tool to generate explainable conformational assumptions and preliminary sorting, rather than a stable quantitative prediction method of combined affinity. In practice, it is usually necessary to adopt a variety of scoring strategies and downstream verification workflows to reduce error propagation (Fig. 1b).

#### Physical realism and computational cost in molecular dynamics

Molecular dynamics simulates the time evolution of molecular complexes in explicit or implicit solvent environments. The resulting trajectory can be used to evaluate structural stability, conformational changes, and key interactions, providing stronger physical support for verifying molecular docking postures and exploring molecular mechanisms. However, many biologically relevant processes occur on microsecond to millisecond timescales. Because traditional molecular dynamics uses a very small time step, it takes a lot of simulation steps to reach such a timescale, and the calculation cost is very high. In addition, a rugged energy landscape will capture the trajectory, resulting in the instability of the results based on finite sampling [24–27]. Enhanced sampling methods can partially alleviate these problems, but they introduce additional parameters, require the selection of reaction coordinates and reweighting procedures, and can add uncertainty. In practice, molecular dynamics remains computationally demanding and typically requires expert setup and careful parameter selection [28, 29] (Fig. 1b).

### The basic principles and potential advantages of quantum computing

#### Basic concepts of quantum computing

Quantum computing is a computing paradigm that uses quantum states to represent and manipulate information. It encodes the information in the quantum bit, and the quantum bit can be in the superposition state of the classical state 0 and 1 before measurement. When multiple quantum bits are combined, the accessible state space will grow rapidly as the number of quantum bits increases. Quantum entanglement can further introduce strong non-classical associations, thus providing computational strategies that are completely different from classical methods for certain types of problems [30–33]. In practice, quantum gates and quantum circuits realize the controlled operation of quantum amplitude and phase, and transform the superposition state and entanglement state into executable algorithm programs [34]. It should be noted that quantum advantage is not universally applicable. Quantum advantage depends on whether the problem structure can be effectively exploited by a quantum algorithm. It also depends on practical factors such as oracle construction, data loading, and measurement cost. Therefore, any claimed acceleration effect must be evaluated through appropriate benchmarking and fair comparison [35, 36]. The core background terms used in this review are summarized in Table 1.

**Table 1:** Core background terms.

Key term	Full name	Definition
NISQ	Noisy Intermediate-Scale Quantum	The current stage of quantum hardware, characterized by device noise, limited qubit counts, and constrained coherence times
Hybrid QC	Hybrid quantum–classical computing	A hybrid computing paradigm in which quantum processors handle a small number of critical computations, while classical computing supports scalable processing and robust validation
QM	Quantum mechanics	Quantum mechanical calculations provide high physical accuracy but are computationally expensive
MM	Molecular mechanics	Classical molecular mechanics and force field models are computationally efficient, but they may be insufficiently accurate for processes such as bond breaking and formation or metal coordination
VQE	Variational quantum eigensolver	High accuracy evaluation and correction of electronic energies for small subsystems
QAOA	Quantum approximate optimization algorithm	Approximate solution of discrete combinatorial optimization subproblems
Rescoring	Ranking rescoring	Refinement and calibration of coarse rankings produced by classical methods
QM/MM	Quantum mechanics/ molecular mechanics	Partitioning of a system into quantum and classical regions to balance accuracy and computational tractability

#### Theoretical advantages of quantum computing

Molecular systems are dominated by quantum mechanics (QM) at the microscopic level. However, high accuracy electronic structure calculations become prohibitively expensive as molecular system size increases, requiring trade-offs between accuracy and computational cost [37, 38]. Quantum computing is therefore often considered to be a natural fit with molecular simulation, because it can directly use quantum bits to represent quantum states and simulate Hamiltonian dynamics through quantum circuits. From the perspective of the calculation framework, this method is closer to the inherent description of multi-body quantum systems, thus providing a theoretical way for calculating molecular energy and properties with higher accuracy. Importantly, this theoretical advantage refers to a potential capability of the computational paradigm rather than guaranteed practical performance. In ideal fault-tolerant quantum computing, quantum algorithms can change the computational complexity and expand the accuracy limits that some core subroutines can achieve [31, 39].

### The scope of this review

In recent years, virtual screening based on deep learning, virtual screening based on molecular docking, and molecular dynamics simulation have become the three most widely used calculation methods in early drug discovery. The deep learning model can quickly prioritize the massive combination library; molecular docking can generate candidate ligand target binding configurations and provide rough sorting; molecular dynamics simulation can evaluate the stability, conformation changes, and binding free energy of complexes. Despite these advances, these three methods still face fundamental bottlenecks in balancing scale, accuracy, and sampling efficiency. The deep learning model is limited by the distribution and generalization ability of training data, the molecular docking is limited by conformational search and simplified scoring functions, and the molecular dynamics is limited by the accuracy of the force field and the challenges of evaluating long-term scale and rare event sampling.

**Figure 2:**
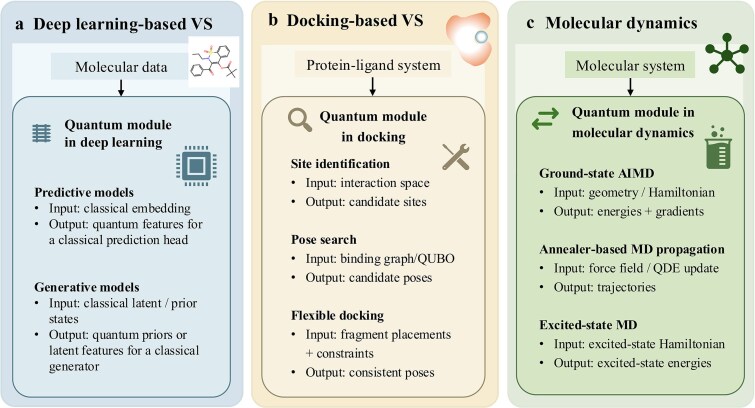
The integration of plugin quantum computing in three typical computational strategies for early drug discovery.

Accordingly, this review focuses on three early stage computational workflows in computer-aided drug discovery: virtual screening using deep learning, virtual screening using molecular docking, and molecular dynamics. Under the Noisy Intermediate-Scale Quantum (NISQ) constraints, it examines quantum computing as a modular addition to classical workflows rather than as a replacement for classical pipelines. This review does not attempt to cover the full drug discovery pipeline or downstream wet lab and clinical validation. As illustrated in Fig. 2, quantum modules may be introduced at selected subroutines within otherwise classical computational procedures. These three workflows are often connected in practical discovery pipelines, but they are not treated here as a compulsory fixed sequence. Rather, each is discussed as an analytically separable workflow in which quantum modules may be introduced independently at selected subroutines when justified by the task and resource budget.

A recent systematic mapping review in Briefings in Bioinformatics by Nałęcz-Charkiewicz *et al*. [40] surveyed quantum computing methods and trends across a wide range of bioinformatics domains. By contrast, the present review focuses specifically on early stage drug discovery. It is organized around three operational computational workflows: virtual screening based on deep learning, virtual screening based on molecular docking, and molecular dynamics, with an emphasis on modular quantum integration within otherwise classical pipelines under realistic NISQ constraints. Within this scope, the review mainly centers on small molecule discovery for protein targets. This focus reflects the current concentration of representative quantum-assisted studies in early stage CADD and allows deep learning, molecular docking, and molecular dynamics to be discussed within a coherent methodological framework. RNA- and DNA-targeted ligand discovery is an important emerging direction, but it is not treated within the workflows discussed in this Review, because our scope focuses on protein-ligand drug discovery and the directly relevant quantum computing literature for RNA- or DNA-targeted ligand discovery remains comparatively limited. In addition to methodological analysis at the workflow level, Table 2 summarizes representative public collaborations between major technology or quantum-focused players and biopharma partners, illustrating the current industrial exploration of quantum approaches in drug discovery at the early stage.

**Table 2:** Representative public collaborations relevant to quantum computing in drug discovery.

Region of primary quantum player	Quantum computing player	Biopharma partner	Collaboration focus
USA	IBM Quantum	Moderna	Public collaboration exploring quantum computing applications in biomedicine and drug discovery within the IBM Quantum Network ecosystem (IBM Newsroom)
China	Tencent Quantum Lab	JOINCARE	Strategic collaboration on quantum computing and AI for synthetic biology and drug discovery (JOINCARE)
USA	SandboxAQ	AstraZeneca	Advancing molecular simulation for drug discovery via the AQBioSim initiative (SandboxAQ)
Switzerland	QC Ware	Roche	Joint exploration of quantum neural networks for drug development tasks (Roche)

## Classical bottlenecks and quantum opportunities in drug discovery

Against this background, this section first outlines the main classical workflows in drug discovery at the early stage and then highlights the bottlenecks that create realistic opportunities for modular quantum integration. Rather than replacing classical systems from end to end, a quantum processor is more plausibly deployed as a coprocessing module for a small number of steps that are especially sensitive, computationally expensive, or decisive. For the purposes of this review, we focus on three bottlenecks specific to these workflows in which modular quantum integration may be particularly relevant: ranking stability in virtual screening based on deep learning, combinatorial search in docking, and local refinement in molecular simulation. Although these areas are often connected in practical discovery workflows, they may also be considered independently, and quantum modules need not be introduced across the full pipeline.

### Deep learning–based virtual screening

In drug discovery, virtual screening based on deep learning typically follows a classical workflow that is well established. First, labeled datasets (such as compound activity, affinity, or phenotypic response pairs) are curated from public databases or internal projects. Molecules are then encoded into representations suitable for modeling, including molecular fingerprints [41], SMILES strings [42], 2D molecular graphs [43], or 3D geometric features [44]. Subsequently, predictive models are trained to perform classification or regression of molecular properties or activities. After model tuning and calibration, the large candidate compound library is screened in batches, scoring is generated, and the compounds are sorted to determine the priority as soon as possible. Only a small part of the top-ranked candidate compounds can enter the stage with higher calculation and experimental requirements, such as molecular docking, free energy calculation, or experimental verification. This hierarchical screening strategy improves the hit rate and reduces the cost of iterative testing.

In virtual screening based on deep learning, the bottleneck often lies in the reliability of ranking rather than the calculation throughput. Label noise, data deviation, distribution offset, and category imbalance will all lead to score instability. Quantum modules are more suitable to provide support at the model level (see Section ‘Classical–quantum hybrid strategies for deep learning models’ for details) [45, 46].

### Virtual screening based on molecular docking

The classic virtual screening based on molecular docking includes extensive conformational sampling, and then screening according to the score. Usually, protein and ligand structures are first prepared by adding hydrogen atoms, assigning charges and protonation states, and defining binding pockets or docking grids [47]. The translation, rotation, and torsional degrees of freedom of the ligand are treated as variables, allowing many candidate poses to be generated across conformational space. Then each candidate pose is scored to roughly estimate the degree of favorability of the combination. The score usually integrates shape complementarity, hydrophobic interaction, hydrogen bond, electrostatic action, and desolvation effect. Promising poses may subsequently be locally optimized or rescored. The dominant computational costs arise from two sources. First, extensive sampling is required in a high-dimensional conformational space [48]. Second, scoring functions must be evaluated repeatedly for each sampled pose. This burden is further amplified in multi-pocket settings, ensemble docking, or large-scale screening [49, 50].

The main difficulty is the combinatorial growth of conformational and pose space in molecular docking-based virtual screening. Docking can be framed as a problem of discrete decisions and combinatorial optimization. Classical docking relies on heuristic search and approximate scoring for efficiency, but it may explore the space insufficiently or get trapped in local optima. Quantum modules are more naturally introduced at selected docking substeps. They can turn a few key discrete decisions into optimization subproblems, produce more choices under a limited call budget, and then return the results to the classical docking procedure for subsequent scoring and assessment [51, 52]. Classical methods provide broad coverage, whereas quantum modules are better suited to supporting a limited number of key decisions (see Section ‘Hybrid classical–quantum docking’ for more details).

### Molecular dynamics

In molecular dynamics [53, 54], the primary task is to simulate atomic motion under a potential energy surface or force field using very small time steps to generate trajectories, from which statistics, such as conformational ensembles, interaction stability, and conformational transitions, can be extracted. The key bottlenecks of classical molecular dynamics fall into two categories—(i) force field accuracy is limited: widely used force fields are approximate and may become unreliable when electronic structure effects are significant, such as polarization, charge or proton transfer, bond breaking and forming, or in systems containing metal centers; (ii) sampling is slow**:** many biologically relevant processes are rare events that occur only on long timescales, causing computational costs to increase rapidly.

In systems involving strong polarization, metal centers, bond changes, or proton transfer, classical force fields can introduce errors into trajectories and free energy estimates. Full-system quantum chemistry is usually too expensive for routine use. A more practical strategy is therefore to use classical molecular dynamics for most of the trajectory, while applying higher accuracy calculations only to selected regions or critical conformations [55, 56]. A small number of high accuracy calls may improve the reliability of key local results. By contrast, directly addressing global sampling bottlenecks with quantum resources remains less mature and is currently harder to justify under NISQ constraints (see Section ‘Hybrid classical–quantum molecular dynamics’ for more details).

## Hybrid classical–quantum computing

### Background

This section focuses on practical quantum computing under NISQ conditions [57–59], at which hardware is limited by noise, limited number of available quantum bits, and limited coherence time. As a result, reliably running quantum algorithms that require deep circuits and full error correction remains challenging. Under these constraints, a more feasible approach is a hybrid quantum–classical paradigm, in which a quantum processor functions as a specialized module within a classical workflow, handling a small number of critical subroutines. Classical computing continues to manage large-scale data processing, candidate generation, and sampling, as well as postprocessing and validation.

At the algorithmic level, NISQ applications typically rely on variational quantum algorithms and hybrid quantum–classical frameworks. In deep learning–based applications, these frameworks are commonly implemented through parameterized quantum circuits, variational quantum classifiers, quantum kernels, and related hybrid learning architectures. Other representative examples include variational quantum eigensolver (VQE) [60] and quantum approximate optimization algorithm (QAOA) [61, 62]. VQE is mainly used for energy estimation and correction in small-scale quantum chemistry and electronic structure problems, while QAOA is mainly used to obtain approximate solutions to discrete combination optimization problems. Since the practical application value of these hybrid strategies depends not only on the algorithm design, but also on the baseline strength, calibration, and affected downstream decision-making points. Table 3 outlines a minimum evaluation template, which will be used to explain the representative research discussed in Sections ‘Classical–quantum hybrid strategies for deep learning models’, ‘Hybrid classical–quantum docking’, and ‘Hybrid classical–quantum molecular dynamics’.

**Table 3:** Minimal evaluation template for quantum-assisted computational strategies in drug discovery.

Workflow	Quantum module	Baseline or comparison	Evaluation output	Resource reporting
Deep learning–based VS	Feature stage augmentation in predictive models; quantum priors or generators in generative models	Fully classical predictor or generator for the same task; matched classical control when feasible	Prediction: AUROC, AUPRC, F1, RMSE, MAE, Pearson, or Spearman. Generation: validity, uniqueness, diversity, novelty, QED, SA, Log P, docking score, or activity	Qubit number, circuit depth, shots, simulator or hardware, noise setting, seeds, runtime
Docking-based VS	Site identification, pose search, and fragment-based flexible docking	Classical site prediction, pose search, graph search, clique search, or fragment placement baseline matched to the selected docking substep	Site accuracy, pose RMSD, binding mode recovery, success probability, ground state overlap, clique recovery, docking configuration quality, or enrichment	Graph or QUBO size, poses or fragments tested, qubits or modes, circuit depth or annealing setting, samples, hardware or simulator, runtime
Molecular dynamics	Ground state AIMD, annealer-based MD propagation, and excited state MD	Classical MD, classical AIMD, QM/MM, or high accuracy reference calculation matched to the quantum-assisted simulation task	Energy error, force error, reference agreement, trajectory stability, structural deviation, vibrational accuracy, excited state agreement, or uncertainty	Subsystem size, atoms or orbitals, corrected frames or states, qubits, circuit depth, shots, measurement strategy, hardware or simulator, runtime

### Classical–quantum hybrid strategies for deep learning models

Under practical NISQ conditions, quantum computing is better positioned as an augmentation module within drug screening and molecular design workflows based on deep learning rather than as a replacement for the full classical deep learning model [63]. In hybrid classical–quantum strategies, classical models handle scalable representation learning and coarse ranking or generation, whereas quantum modules are applied only to selected submodules under feasible resource limits. With evaluation criteria at the workflow level outlined in Table 3, related representative study designs, quantum components, classical comparators, and operational roles are summarized in Table 4, while indicative resource feasibility and data provenance, endpoints, and realism caveats are summarized in Tables 6 and 7.

**Table 4:** Representative quantum-assisted drug discovery studies and their practical evaluation context.

ID	Refs.	Task	Quantum component	Classical comparator(s)	Stage of use and metrics	Scope notes
1	[64]	Affinity prediction	Hybrid quantum module inserted after classical feature encoding within HQDeepDTAF	DeepDTAF; DeepDTA; Pafnucy; TopologyNet; AEScore; KDEEP	Prioritization before experimental validation; MAE, RMSE, Pearson, SD, and CI	Retrospective benchmark level affinity regression study; no wet lab validation and no calibration analysis
2	[65]	Affinity prediction	Hybrid quantum layers in GIN, GCN, and CNN models	GIN, GCN, and CNN; also ML-DTI and Yuel	Screening support; Pearson, Spearman, and RMSE; downstream support evaluated with MM/GBSA, MM/PBSA, and MD	Affinity prediction support setting with downstream MM/GBSA, MM/PBSA, and MD checks; still benchmark scale rather than a full prospective screening workflow
3	[66]	Affinity prediction	Hybrid quantum fusion model combining 3D-CNN and SG-CNN with a QNN	Classical fusion model; prior classical 3D-CNN and SG-CNN approaches	Screening support; R^2^, MAE, MSE, Pearson, and Spearman	Affinity prediction benchmark based on structural information; useful for screening support, but still retrospective and not a complete workflow for screening large libraries
4	[67]	Property prediction	QNEM + QEEM within hybrid QEGNN	Classical GCN, CCN, GIN, and GAT	Prioritization before experimental validation; accuracy and loss were reported	Retrospective molecular property benchmark using standardized or balanced subsets; results may not reflect screening imbalance or deployment conditions in real settings
5	[68]	KRAS inhibitor generation	QCBM quantum prior + LSTM generator + Chemistry42 reward/filter	REINVENT; SMILES-/SELFIES-VAE; MoFlow; SMILES-/SELFIES-LSTM-HC; GB-GA; JANUS; vanilla LSTM	Triage before synthesis; Tartarus success rate, docking score, and protein ligand interaction ranking; 1 M compounds per model; top 15 compounds synthesized; SPR and cell assays	Hybrid workflow from molecular generation to experimental validation with synthesis and assay confirmation, but still biased toward known KRAS chemistry and docking-based proxies; no definitive quantum advantage established
6	[69]	Molecule generation	Hybrid quantum generator and hybrid quantum cycle component in MolGAN	MolGAN; Cycle-MolGAN; prior hybrid quantum GAN models	Molecule generation; QED, SA, Log P, validity, uniqueness, and diversity	Benchmark scale small molecule generation on QM9/PC9 using proxy molecular metrics; validation remained mainly simulator-based, with no experimental follow-up in drug discovery
7	[70]	Docking site identification	Modified Grover search on a protein interaction space representation, with quantum search and site evaluation modules	No direct comparison with standard classical tools for binding site prediction was reported	Binding site localization before docking identification demonstrated on a quantum simulator and a real quantum computer	Binding site localization before docking rather than full prediction of docking poses; simplified encoding of interaction space with only selected interaction types considered
8	[51]	Docking pose search	QAOA/DCQAOA for optimization of the maximum weight clique in the protein ligand interaction graph	Conventional QAOA; exact diagonalization; PLIP consistency check	Pose selection; success probability and overlap with the ground state	Proof of concept docking pose selection on a very small set of protein ligand complexes; no large library screening or re-ranking evaluation at the candidate level
9	[71]	Docking pose sampling	Gaussian Boson Sampling (GBS) for maximum weight clique search in a binding interaction graph	Classical random search; greedy shrinking; local search	Configuration search; success rate for maximum weight clique recovery and correct binding mode recovery	Proof of principle study for docking configuration search in a simplified interaction graph; strongly dependent on graph construction and not a complete docking benchmark
10	[72]	Docking pose search	GBS on a binding interaction graph reduced to a maximum weighted clique problem	Classical postprocessing/reconstruction steps are used, but no full classical docking baseline is replaced	Docking configuration search; predicted docking configurations are selected by clique weight; benchmarked on a known ligand receptor interaction rather than standard metrics for large-scale screening	Uses a simplified graph representation based on pharmacophores and numerical simulation; explicitly improves configuration sampling rather than the main difficulty of scoring
11	[73]	Flexible docking	Fragment-based QUBO docking with clash, bond, and single placement constraints; solved by SQBM+	Classical fragment placement + downstream energy minimization; no direct flexible docking benchmark reported	Docking configuration search; predicted docking configurations are selected by clique weight; benchmarked on a known ligand receptor interaction rather than standard metrics for large-scale screening	Proof of concept re-docking on aldose reductase; more realistic than rigid matching because internal degrees of freedom are represented through fragment consistency, but still based on simulation
12	[74]	Ground state AIMD	VQE electronic energies with numerical gradients via HFT + correlated sampling	Classical AIMD formulation; exact/FCI references for validation	Ground state AIMD; proof of concept H_2_ trajectories on IBM devices; energies and gradients are central	Proof of concept for small systems; larger systems were assessed mainly through classical reference wave functions
13	[75]	Annealer assisted MD propagation	QDE framework using a D Wave quantum annealer, followed by classical greedy postprocessing	Analytical reference solution; classical greedy postprocessing	MD propagation; H_2_ vibrational trajectories in harmonic, anharmonic, and dissociative regimes; trajectory accuracy/convergence central	Proof of principle for very small systems; focuses on trajectory propagation rather than electronic structure calculation
14	[76]	Excited state MD	Restricted VQE excited state MD with SSVQE/VQST state preparation	Exact adiabatic S1 simulations as reference	Excited state MD; S1 dynamics of H_2_ and CH_2_NH; agreement with exact adiabatic results except near conical intersection	Proof of concept for small molecules; effectively diabatic near conical intersections; not yet practical for large-scale MD

Integration at the model level uses quantum modules within otherwise classical learning models to enhance selected computational stages (Fig. 2a). In the drug discovery literature reviewed here, such integration has mainly appeared in two broad forms, while related representative study designs, quantum components, classical comparators, and operational roles across the reviewed studies are summarized in Table 4. The first comprises predictive models. Within this group, the main pattern is to introduce quantum modules at the representation or feature stage. For example, Jeong *et al*. [64] introduced a hybrid quantum neural network after classical feature encoding, while related hybrid affinity models [65, 66] similarly introduced small quantum submodules after classical encoders or feature fusion stages for binding affinity prediction. This predictive form also includes quantum embedded graph models for molecular property prediction, as illustrated by quantum embedded graph neural network (QEGNN) [67], where a quantum module is coupled to graph-based molecular representations. The second broad form comprises generative molecular design models, in which quantum components act as priors or generators, including generation of Kirsten rat sarcoma viral oncogene homolog (KRAS) inhibitor generation using a quantum circuit Born machine (QCBM) prior [68] and hybrid quantum cycle molecular generative adversarial network (HQ-Cycle-MolGAN) for small molecule generation [69].

Although task categories and model architectures vary, these studies show similar integration patterns: quantum components are inserted into specific submodules of classical deep learning models instead of replacing the entire predictor or generator. However, the current evidence is still uneven between different tasks and assessment settings. Most studies are still based on simulation, limited hardware verification, or benchmark test level evaluation. Only a few studies progress to prospective screening. The study of KRAS inhibitors is a significant exception, because it has developed from the computational generation stage to the stage of compound synthesis and experimental verification.

### Hybrid classical–quantum docking

Under the constraints of NISQ, the more realistic quantum participation docking method is not to replace the entire docking software stack, but to use quantum computing as a modular plugin to handle selected sensitive and controllable docking steps. Classical computing continues to be responsible for pose generation, geometric constraint processing, and large-scale enumeration and rough scoring. Quantum modules only intervene in specific stages, such as position recognition, pose search, or fragment-based flexible docking. Their role is to support selected docking-related subproblems under a limited quantum call budget, rather than to replace the full docking workflow [56, 63]. The notes on the relevant representative research design, resource feasibility, and reality are summarized in Tables 5–7.

**Table 5:** Indicative resource feasibility for representative quantum use cases.

ID	Refs.	Use case	Quantum scale	Circuit depth	Shots	Error mitigation	Setting	Practical caveat
1	[64]	Affinity prediction	7–9 qubits; 9 qubits in the main configuration	20 layers; reported cost ~260 versus ~10 480 in the compared setting	Not reported	No explicit QEM; future QEM was suggested	Classical simulation only (PennyLane/PyTorch); no real hardware	Among the tested settings, NN angle appears the most plausible under NISQ conditions, although performance degrades under noise
2	[65]	Affinity prediction	6 qubits in graph layers; 10 qubits after drug–protein fusion	PQC with m entanglement layers followed by one single qubit layer; exact m deferred to SI	Not reported	Not reported	Classically simulated PQC-based hybrid deep learning	No real hardware execution; noise, error mitigation, and hardware feasibility not analysed
3	[66]	Fusion affinity prediction	4 qubits (amplitude) or 8 qubits (hybrid angle)	PQC Circuit 1, L = 10	Not reported	DREM was effective mainly for *P* ≤ .05	Classical simulation only; no execution on actual quantum hardware reported	Simulation only setting; DREM appears effective mainly under low noise
4	[67]	Property prediction	4–9 qubits; validated on 72 qubit Wukong hardware	Shallow HE-VQC; about 4 CZ layers	Not reported	Not reported	Simulator + Wukong hardware validation	Validation on real hardware was reported, but only on small and standardized datasets
5	[68]	KRAS generation	16 qubits	4-layer QCBM	Not reported	Mentioned, but not specified	IBM Guadalupe + classical baselines	Early experimental validation, but small synthesis scale, micromolar activity, and no definitive quantum advantage
6	[69]	Molecule generation	8 qubits; 2 ancilla	3 variational layers +8 encoding layers	2 × 10^5^ shots	No explicit QEM; only comparison between noisy and ideal simulators	Classical training followed by noisy and ideal IBM Brisbane simulator runs; no execution on real quantum hardware	Validation was mostly based on simulation; the reported high entropy state does not by itself establish a stable practical advantage
								
7	[70]	Docking site identification	2 qubits per interaction site; total size depends on the encoding of ligand and protein interaction space	Modified Grover search combined with a site evaluation circuit; depth not reported	Not reported	Not reported	Qiskit simulator + real quantum computer validation	Upstream site identification only; simplified interaction space encoding using selected interaction types
8	[51]	Docking pose selection	6–12 qubits	QAOA/DCQAOA with p layers; p = 8 worked best in the DCQAOA case using 8 qubits	5000 samples; 500 trials	No explicit QEM was used; the analysis only considered depolarizing noise	MindQuantum simulation only	Useful for small docking subproblems, but challenges in scaling and local optima remain
9	[71]	Docking graph search	24 optical modes	Photonic GBS; qubit gate depth is not applicable	10^5^ and 10^4^ samples	No explicit QEM; robustness to photon loss was discussed	Photonic simulation using a simplified coarse-grained graph	Supports docking configuration search rather than full scoring function evaluation
10	[72]	Docking pose search	Photonic GBS; optical modes scale with the size of the binding interaction graph, with 24 interaction pairs in the benchmark case	Gate circuit depth is not applicable; Gaussian optical network	Not reported	No explicit QEM; robustness to photon loss discussed	Numerical photonic simulation; no execution on real hardware in this study	Improves sampling of docking configurations in a simplified pharmacophore graph; does not address the main difficulty of scoring function evaluation
11	[73]	Flexible docking	QUBO size depends on the fragment placement problem; solved by SQBM+ simulated quantum annealer	Based on annealing; no gate circuit depth reported	Not reported	Not reported	Proof of concept re-docking using SQBM+ simulation followed by classical energy minimization	More realistic than rigid matching because internal degrees of freedom are represented through fragment consistency, but validation remains based on simulation
12	[74]	Ground state AIMD	H_2_ mapped from 4 qubits to 2, and further to 1 qubit	UCCSD circuit; H_2_ implementations using 1 and 2 qubits	295 × 10^3^ on IBM Q Vigo	Measurement error mitigation (Qiskit Ignis)	IBM Q Vigo hardware + state vector simulation	Small H_2_ proof of concept; gradients are numerical rather than fully quantum-analytic; larger systems assessed mainly via classical validation
13	[75]	MD propagation based on an annealer	D-Wave 2000Q; 2048 physical qubits, up to 64 fully connected logical qubits	QUBO formulation with an annealing setting; gate circuit depth is not applicable	Not reported	No explicit QEM was used; the best accuracy was obtained after classical greedy postprocessing	Real D-Wave 2000Q annealer	H_2_ vibrational dynamics only; proof-of-principle trajectory propagation, not electronic structure MD
14	[76]	Excited state MD	4 qubits for H_2_; 6 qubits for CH_2_NH	HE ansatz; SSVQE depth > 4 for H_2_ and > 12 for CH_2_NH; shallower VQST states also used	Not reported	No explicit QEM; measurement partitioning on commuting observables	Sampling simulations and runs on the IBM Kawasaki Falcon device	Proof of concept for small molecules; effectively diabatic near conical intersections

**Table 6:** Data provenance, supervision targets, and bias/realism caveats in representative quantum-assisted drug discovery studies.

ID	Refs.	Task	Data source/benchmark/scale/split	Supervision target/endpoint	Potential bias/realism caveat
1	[64]	Affinity prediction	PDBbind v2016 core benchmark; protein/pocket structures in PDB format and ligands represented by SDF/SMILES; core 2016 used for evaluation; fixed token lengths 1000/63/150	Supervised regression: −log(Ki/Kd/IC50)	Retrospective benchmark only; no wet lab validation; NISQ feasibility was assessed mainly through simulation, without demonstrated deployment on a real QPU
2	[65]	Affinity prediction	Davis, Metz, KIBA, and PDBbind benchmarks with totals of 31 824/35 259/160 278/13 196; reported train and test splits with five-fold cross validation; plus a liver disease screening set of 1500 pairs for application use	Supervised regression for protein ligand binding affinity; labels derived from Kd, Ki, and KIBA; evaluated by Pearson, Spearman, and RMSE	Retrospective benchmark study with quantum layers evaluated only by simulation; no validation on real devices or in wet lab experiments; downstream screening was checked mainly by MM/GBSA, MM/PBSA, and MD
3	[66]	Affinity prediction	PDBbind 2020; refined set 5316 for training/validation and core set 285 for testing; 25% of the refined set used for validation	Supervised regression: binding affinity, evaluated by R^2^, MAE, Pearson, Spearman, and MSE	Validation based on simulation rather than real devices; DREM mitigation was shown mainly at *P* ≤ .05
4	[67]	Property prediction	ClinTox, HIV, and BACE; original sizes 1491, >40 k, and 1522; standardized to 500 each; 80:20 train/test split	Supervised classification: molecular property, activity, or toxicity label	Downsampling and label balancing reduce real world realism; still retrospective, although also tested on Wukong hardware
5	[68]	KRAS generation	~650 known KRAS inhibitors; 850 k STONED-SELFIES analogs; top 250 k from 100 M Enamine REAL; ~1.1 M total; 1 M generated compounds screened; 15 compounds synthesized; no explicit split	Goal-directed molecular generation using Chemistry42 and local filters, followed by docking and protein ligand interaction ranking; endpoints included SPR, MaMTH DS IC50/activity, and cell viability	Biased toward known KRAS chemistry and docking proxies; only 15 compounds were tested; still early stage hits; no definitive quantum advantage established
6	[69]	Molecule generation	QM9 (~134 k) and PC9 (~99 k); single or combined dataset training; small validation sets (100/250); no standard split emphasis	Goal-directed molecular generation using Chemistry42 and local filters, followed by docking and protein ligand interaction ranking; endpoints included SPR, MaMTH DS IC50/activity, and cell viability	Small validation sets; optimization based on proxy metrics; reported high entropy state; hardware assessment relied mainly on noisy simulation
7	[70]	Docking site identification	Representation of protein ligand interaction space with validation on both simulator and real quantum computer; no standard benchmark split reported	No supervised target; endpoint: identification of candidate docking sites	Simplified lattice/interaction space encoding; only selected interaction types were included, and the study addresses upstream site localization rather than full docking pose prediction
8	[51]	Docking pose selection	Three case studies: 8SKH, 3HAC, and 5F4L; reduced to 6-, 8-, and 12-qubit BIG/QUBO instances; no train/test split	No supervised target; endpoint was the optimal docking pose or the maximum weight clique	Relies on substantial pharmacophore pruning and heuristics; one case used proximity to the actual binding pose for point selection; scaling remains limited by local optima and parameter growth
9	[71]	Docking search	Case study of a known TACE aryl sulfonamide interaction; coarse-grained graph with 4 ligand nodes and 6 receptor nodes, giving 24 interaction pairs; no train and test split reported	No supervised target; endpoint was the maximum weight clique or docking configuration	Does not address the limitations of scoring function evaluation; relies on a simplified coarse-grained representation; some benchmarking was performed after fixing the clique size
10	[72]	Docking pose search	Known interaction between TACE and aryl sulfonamide; coarse-grained pharmacophore graph with 4 ligand nodes and 6 receptor nodes, giving 24 interaction pairs; no train and test split reported	No supervised target; endpoint: maximum weighted clique/docking configuration search	Uses a simplified pharmacophore representation and numerical simulation; improves configuration sampling rather than addressing the main difficulty of scoring function evaluation; some benchmarking was performed after fixing the clique size
11	[73]	Flexible docking	Aldose reductase, ALDR, PDB ID: 2HV5; proof of concept re-docking using 343 subregions and 3005 candidate fragment placements; no train and test split reported	No supervised target; endpoint: reconstructed binding pose/re-docking RMSD	Single target proof of concept using SQBM+ simulated quantum annealer; realism is limited by fragment decomposition and a manually defined docking region, although internal degrees of freedom are better represented than in rigid formulations
12	[74]	Ground state AIMD	Proof-of-concept AIMD study for H_2_ on IBM quantum devices; larger molecules were assessed using classical FCI wave functions; no train and test split reported	No supervised target; endpoint: AIMD trajectory propagation from VQE energies and numerical gradients	Proof of concept for very small systems; real hardware MD was shown only for H_2_, while relevance to larger systems was assessed mainly through classical validation
13	[75]	MD propagation based on an annealer	H_2_ vibrational dynamics on D-Wave 2000Q; nearly harmonic, highly anharmonic, and dissociative regimes; no train/test split	No supervised target; endpoint: trajectory propagation/convergence to analytical reference solution	Proof of principle study using a diatomic molecule; focuses on trajectory propagation rather than electronic structure MD, with results improved by hybrid classical postprocessing
14	[76]	Excited state MD	Examples of S1 excited state MD for H_2_ and CH_2_NH small molecules; no train and test split reported	No supervised target; endpoint: excited state MD trajectories compared with exact adiabatic S1 simulations	Small molecule proof of concept; agreement degrades near conical intersection, where the method becomes effectively diabatic rather than fully adiabatic

**Table 7:** Abbreviations used in this manuscript.

Abbreviation	Full name
ADMET	Absorption, distribution, metabolism, excretion, and toxicity
BACE	β-site amyloid precursor protein cleaving enzyme
CADD	Computer-aided drug design
CASCI	Complete active space configuration interaction
CNN	Convolutional neural network
DFT	Density functional theory
DTI	Drug–target interaction
GAT	Graph attention network
GCN	Graph convolutional network
GIN	Graph isomorphism network
HF	Hartree–Fock
HIV	Human immunodeficiency virus
HQNN	Hybrid quantum neural network
IBM	International Business Machines
IC50	Half maximal inhibitory concentration
Kd	Dissociation constant
Ki	Inhibition constant
LogP	Octanol/water partition coefficient
LSTM	Long short-term memory
MAE	Mean absolute error
MD	Molecular dynamics
MM	Molecular mechanics
MM/GBSA	Molecular mechanics/generalized Born surface area
MM/PBSA	Molecular mechanics/Poisson–Boltzmann surface area
MSE	Mean squared error
NISQ	Noisy Intermediate-Scale Quantum
PDB	Protein Data Bank
PDBbind	Protein Data Bank binding database/benchmark set
PQC	Parameterized quantum circuit
QAOA	Quantum approximate optimization algorithm
QCBM	Quantum circuit born machine
QED	Quantitative estimate of drug-likeness
QM	Quantum mechanics
QM/MM	Quantum mechanics/molecular mechanics
QNN	Quantum neural network
QPU	Quantum processing unit
QSVM	Quantum support vector machine
R	Pearson correlation coefficient
R^2^	Coefficient of determination
RMSE	Root mean squared error
SA	Synthetic accessibility
SELFIES	SELF-referencing embedded strings
SMILES	Simplified molecular input line entry system
SPR	Surface plasmon resonance
VAE	Variational autoencoder
VQE	Variational quantum eigensolver
VS	Virtual screening
AIMD	*Ab initio* molecular dynamics
GBS	Gaussian Boson sampling
QUBO	Quadratic unconstrained binary optimization
QEM	Quantum error mitigation
UCCSD	Unitary coupled cluster with singles and doubles
SSVQE	Subspace search variational quantum eigensolver
VQST	Variational quantum state transcription
HE ansatz	Hardware efficient ansatz
HE-VQC	Hardware efficient variational quantum circuit
PLIP	Protein ligand interaction profiler
SD	Standard deviation
CI	Confidence interval

In the molecular docking literature reviewed in this article, quantum integration mainly appears in three forms: site recognition, pose search, and flexible docking (Fig. 2b). In site identification, quantum search is used to locate candidate ligand binding regions before full pose generation. For example, in the research of Liliopoulos *et al*. [70], they applied the improved Grover search to the spatial representation of protein interaction. In posture search, molecular docking is re-expressed as graph search, group search, or matching problem to identify reasonable docking configuration. Representative examples include Banchi *et al*. [71] using Gaussian boson sampling for maximum weighted group search; Ding *et al*. [51] using quantum approximate optimization algorithm (QAOA) and digitized counterdiabatic QAOA (DCQAOA) in the combined interaction diagram; and Zha *et al*. [72] using grid point matching and characteristic atom matching to collect posture. The sample is coded as a quadratic unconstrained binary optimization (QUBO) instance. In flexible docking, fragment-based models begin to consider the internal degree of freedom of the ligand. For example, Yanagisawa *et al*. [73] formulated protein ligand flexible docking as a QUBO problem by incorporating protein fragment interactions, fragment clashes, covalent connectivity, and single placement constraints.

Although there are differences in the definition of tasks, the reviewed studies follow a common practical logic: quantum modules are applied to specific subproblems related to docking, not the complete docking workflow. Current presentations are still limited in terms of scale and authenticity, and usually rely on simplified representations, proof-of-concept settings, or validation based on simulation.

### Hybrid classical–quantum molecular dynamics

Under the constraints of NISQ, the real path of quantum computing in molecular dynamics does not replace the entire molecular dynamics process, but provides electronic structure level correction for specific areas where the classical force field is not reliable. Classical engines remain responsible for trajectory propagation, thermostat and barostat control, and long timescale sampling. Quantum modules are instead most relevant to local regions involving bond formation or breaking, metal coordination, proton transfer, or strong polarization, where fixed topology or fixed charge force fields may fail. In such settings, a quantum processor could be used to estimate local electronic energies, forces, or interaction components, which are then passed back to the classical molecular dynamics or QM/molecular mechanics framework. Current demonstrations remain proof of concept and are still limited to small systems, but they clarify the intended division of labor: classical computing provides scale and sampling, whereas quantum computing is used to improve the accuracy of selected electronically complex regions [77].

In the molecular dynamics literature reviewed here, representative quantum participation has mainly appeared in three forms: ground state *ab initio* molecular dynamics, molecular dynamics propagation based on annealers, and excited state molecular dynamics (Fig. 2c). The first form is ground state *ab initio* molecular dynamics. Fedorov *et al*. [74] used VQE to compute electronic energies on a quantum computer and propagated nuclei classically on the resulting potential energy surface. This enabled proof-of-concept *ab initio* molecular dynamics simulations of H_2_ on IBM quantum devices, with gradients evaluated numerically. The second form is based on the molecular dynamics propagation of the annealer. As Gaidai *et al*. [75] said, they developed a quantum differential equation framework and used the D-Wave 2000Q quantum annealer to propagate the vibration trajectory of H_2_. The third form is excited state molecular dynamics. Hirai [76] proposed an excited state molecular dynamics method based on variational quantum algorithms and demonstrated S1 state dynamics for H_2_ and CH_2_NH. In these studies, quantum components enter the molecular dynamics cycle at the level of energy evaluation, calculations related to forces, or trajectory updates, and the broader simulation workflow remains hybrid rather than completely quantum.

Accordingly, under current NISQ constraints, the most practical role of quantum computing in molecular dynamics is selective local correction or targeted trajectory assistance within an otherwise classical sampling framework. Rather than being used for full-system sampling, it provides high accuracy electronic structure and energy information for a localized key region or supports the propagation of simplified trajectories or excited state dynamics in small systems, which is then fed back into the classical molecular dynamics loop. Given current limitations in qubit count, noise, and measurement overhead, practical implementations require strict control over subsystem size and the frequency of quantum calls. Consequently, a realistic engineering strategy is to call the quantum module only at a limited number of critical conformational states or time points, or to restrict it to small model systems and proof-of-concept dynamical settings, while leaving most trajectory propagation to classical force fields [78, 79].

## Challenges and outlook

### Current major challenges

Although the preceding sections describe practical strategies for embedding quantum modules into deep learning–based virtual screening, molecular docking-based virtual screening, and molecular dynamics, several key hurdles remain before these approaches can advance beyond the proof-of-concept stage and become reproducible, comparable, and deployable hybrid workflows. These challenges can be broadly grouped into four categories: hardware and noise constraints, interface alignment, benchmarking and reproducibility, and relevance to decision points, as described below:

**(1) Hardware and noise constraints**

Gate errors, decoherence, and measurement noise on NISQ devices require quantum modules to remain shallow, measurement-light, and sparingly invoked; otherwise, output variance and operational costs grow rapidly, undermining reproducibility, and obscuring marginal gains [80, 81].

**(2) Interface alignment**

The mismatch between molecular inputs, quantum representations, and classical outputs remains a central issue. High-dimensional molecules and protein representations usually have to be compressed into low-dimensional forms that can be processed by quantum devices. In turn, quantum outputs (such as learned features or energy correction) must be aligned with classical fractions in scale and statistical interpretation to achieve interpretable fusion, calibration, and reliable reordering [82].

**(3) Benchmark test and repeatability**

The reliability of hybrid methods depends on whether they are superior to the strong classical baseline under strict experimental conditions and whether they can withstand the effects of random seed and sampling noise (see Table 3). In addition to the overall indicators, the evaluation should also emphasize early enrichment, sorting stability, and calibration, while clearly reporting the circuit depth, number of samples, and optimizer settings [83].

**(4) Relevance with decision-making points**

Even if the hybrid method shows measurable improvements in benchmark indicators, it does not automatically prove that it is reasonable to use quantum integration in practice. Its value ultimately depends on whether the gain is large enough to influence downstream decision-making points under the available runtime and quantum call budget, not just improve intermediate indicators [59, 63].

In order to meet these practical challenges, Table 3 provides a minimum evaluation template for the drug discovery workflow based on quantum computing, which clarifies the expected baseline, calibration indicators, and virtual screening based on deep learning, virtual screening based on molecular docking, and virtual screening of molecular dynamics under the constraints of running time. Table 4 summarizes a practical evaluation and reporting framework suitable for the typical study of quantum-assisted drug discovery, including the workflow stage, quantum action, classical baseline, decision-making impact, and benchmarking background. In addition, Table 5 summarizes the indicative dimensions of resource feasibility in typical use cases, including quantum bit size, circuit depth, number of experiments, error mitigation hypothesis, and hardware or analog settings. Table 6 further summarizes the data provenance, supervision targets, and caveats related to bias and realism in representative studies discussed in this review. Box 1 summarizes these practical requirements as a minimal benchmarking checklist for studies of quantum-assisted drug discovery. Together, these materials highlight that the key barrier is not whether a quantum module can be inserted into a workflow in principle, but whether it can deliver reproducible gains relevant to decisions under realistic NISQ constraints.

**Box 1. Minimal benchmarking checklist for quantum-assisted drug discovery studies**

- **Strong classical baselines**. Compare against relevant classical baselines at the same workflow stage rather than against weak or mismatched references only.
- **Multiple random seeds**. Report variability across repeated runs; for stochastic quantum components, separate seed effects from shot noise effects when possible.
- **Sensitivity analysis for shot noise**. Evaluate whether conclusions remain stable under different shot budgets or realistic sampling noise.
- **Ablation studies**. Distinguish gains from the quantum module itself from gains due to feature fusion, re-ranking strategy, workflow redesign, or other auxiliary components.
- **Resource reporting**. Explicitly report qubit count, circuit depth, shot budget, optimizer, and assumptions for error mitigation.
- **Decision relevance**. State the exact decision point affected by the quantum module and the corresponding runtime budget or intervention scope.

### Conclusions and future outlook

In the foreseeable NISQ era, quantum computing in drug discovery is most appropriately framed as a coprocessor plugin, and its value lies in delivering verifiable marginal gains for a small number of critical subroutines, rather than replacing classical pipelines end-to-end. In many discovery workflows in the real world, computational ranking still needs to support difficult prioritization decisions at a late stage before experimental validation, reflecting the fact that current methods do not always provide sufficient confidence for final selection. From this perspective, a meaningful practical test for quantum computing is not merely whether it improves benchmark metrics, but whether it can improve ranking reliability and decision confidence at these important decision points.

Across deep learning–based virtual screening, molecular docking-based virtual screening, and molecular dynamics, practical hybrid strategies are beginning to emerge. Classical computing continues to handle scalable representation learning, pose generation, coarse scoring, trajectory propagation, sampling, and robust validation, while quantum modules intervene either within predictive or generative deep learning models, or at selected substeps in docking and molecular dynamics. In the representative studies reviewed here, these roles include augmentation at the representation or feature stage and quantum priors or generators in deep learning; site identification, pose search, and flexible docking based on fragments in docking; and ground state *ab initio* molecular dynamics, molecular dynamics propagation based on annealers, and excited state molecular dynamics in molecular dynamics.

Looking ahead, a realistic path for quantum computing in drug discovery is to remain within hybrid workflows and focus on a small set of decision-sensitive and computationally intensive steps. The goal is not to replace the full pipeline with quantum computing, but to achieve targeted improvements within hybrid workflows. Progress should therefore be judged by whether quantum modules deliver reproducible gains at specific downstream decision points under realistic runtime and resource constraints.

**Key Points**

- In the Noisy Intermediate-Scale Quantum (NISQ) era, quantum computing in drug discovery is best developed as a modular coprocessor for selected subroutines that are sensitive for decisions, rather than as a replacement for full classical workflows.
- In screening and design based on deep learning, the most plausible roles of quantum modules in the near term are augmentation at the representation or feature stage in predictive models and the use of quantum priors or generators in molecular design models.
- In virtual screening based on docking, realistic quantum roles are concentrated at selected substeps related to docking, especially site identification, pose search, and flexible docking based on fragments, rather than full docking replacement.
- In molecular dynamics, current representative quantum participation spans ground state *ab initio* molecular dynamics, trajectory propagation based on annealers, and excited state molecular dynamics, while most sampling at a large-scale remains classical.
- Quantum computing should be evaluated not only by benchmark accuracy, but also by decision relevance, ranking and calibration reliability, and comparison with strong classical baselines under explicit runtime and resource constraints.
- In order to ensure the repeatability and comparability of the research results, the report should clearly explain the quantum bit scale, circuit depth, experimental budget, error mitigation hypothesis, optimizer settings, and the specific position and frequency of quantum calls in the workflow.

## Data Availability

No new data was generated or analysed in this work.
